# Study on physical properties of four pH responsive *Spodoptera exigua* multiple nucleopolyhedrovirus (SeMNPV) microcapsules as controlled release carriers

**DOI:** 10.1038/s41598-022-26317-5

**Published:** 2022-12-19

**Authors:** Meng Luo, Juntao Lin, Xinhua Zhou, Xia Pu

**Affiliations:** Key Laboratory of Agricultural Green Fine Chemicals of Guangdong Higher Education Institution, School of Chemistry and Chemical Engineering, Zhongkai University of Agriculture and Engineering, Guangzhou, 510225 People’s Republic of China

**Keywords:** Natural hazards, Biomaterials

## Abstract

This study provides a promising controlled release form of nuclear polyhedrosis virus (NPV) for targeted control of lepidopteran pests. However, the application of NPV is limited due to its sensitivity to UV inactivation. This study investigated the anti-UV properties of microcapsules of SeMNPV occlusion bodies (OBs) encapsulated by calcium alginate (CA), and also the influence of the modification of CA by chitosan (CS), whey protein (WP), and polydopamine (PDA). These capsules were used to deliver, in a controlled release manner virions under alkaline pH conditions. Characterization of the structure, morphology, particle size, encapsulation efficiency, contact angle, insecticidal activity, UV resistance and in vitro release of the microcapsules was conducted. The modified microcapsules had better sphericity, and were devoid of SeMNPV OBs on the surface. The encapsulation rate was 84.76 ± 0.59%. PDA@CA-NPV had the highest wettability and the contact angle was 74.51 ± 0.53°. The 50% lethal concentration values (LC_50_) of CA-NPV, CS@CA-NPV, WP@CA-NPV and PDA@CA-NPV were 11.5, 10.7, 10.5 and 1.2 times that of SeMNPV OBs alone. The modified microcapsules all improved the anti-UV performance of the virus, and PDA@CA-NPV was the most UV-resistant. Using qPCR, it was observed that under alkaline conditions, a large number of virions were released from PDA@CA-NPV, CA-NPV and SeMNPV OBs. Microencapsulated virus under alkaline conditions did not change the release pattern of virions.

## Introduction

The *Spodoptera exigua* multiple nucleopolyhedrovirus is a highly pathogenic host-specific alphabaculovirus that has been used as a biological insecticide to control *S. exigua* (Lepidoptera: Noctuidae) populations^1,2^. Exposure to solar ultraviolet radiation (UVB, 280–320 nm) has been a limiting factor in the persistence of entomopathogenic viruses^3^. Addressing this problem, microencapsulation is a method that can be used for providing effective protection of pathogens from UV light^4^.

Natural polymers have been the most popular class of material studied for microencapsulation because of their biocompatibility, reduced toxicity, and easy processing. Some polymers, such as lignin and Eudragits100 have been used for the microencapsulation of viruses^5–7^. Lignin microcapsules, made by spray drying, also enhanced the photostability of the insect virus, but the lignin shell of the microcapsules dissolved in aqueous solution within hours. The significance of maintaining an UV protective layer around the virus after spraying was lost. Anagrapha falcifera nucleopolyhedrovirus in lignin-based spray-dried formulations had a longer shelf-life and residual insecticidal activity in the field compared with the non-encapsulated formulation^8^.

Lepidopteran larvae have an alkaline gut with a pH as high as 12^9,10^. This characteristic could potentially be exploited for the targeted pest control of lepidopteran pests^11^. The development of intelligent microcapsules with pH-controlled release systems is a research hot-spot in pesticide formulation^12,13^, but rarely in insects^14^.

Alkaline pH-responsive drug delivery systems usually have acidic groups such as microcapsules with a carboxyl group as the response factor. The side chain of the carboxyl group on the polymer segment becomes deprotonated, leading to an increase in hydrophilicity. Under alkaline conditions, a decrease in the hydrophobic ratio emerges, resulting in destruction of the microcapsule structure, followed by drug release^15^.

One such delivery system uses sodium alginate (SA), a water-soluble, natural polysaccharide–based, pH-responsive anionic polymer^16^. However, pure alginate beads show large network porosity, loose structure, and poor mechanical strength, resulting in poor embedding efficiency and controlled release of a pesticide. The stability of alginate beads can be enhanced by the interaction between polysaccharides and protein^17–19^.

Chitosan is a polycationic linear copolymer of b-(1–4)-linked N-acetylglucosamine and glucosamine^20^. Whey protein is commonly used as an emulsifier to stabilize oil-in-water preparations^21,22^. This protein has an isoelectric point (pI) of around pH 5, and tends to be positively charged below this value^23^. The two positively charged materials and hydroxyl groups on alginate beads have been shown to modify the structure of the beads as a whole. Therefore, it is expected to enhance its resistance to UV light. Polydopamine is a kind of adhesive biomaterial that is formed by self-polymerization of dopamine under weak alkali conditions^24–27^. The presence of catechol and amino groups in the structure of dopamine can self-oxidize and polymerize on almost all solid surfaces to form polydopamine coatings or polydopamine microcapsules^28–31^. Polydopamine coatings containing a large number of phenolic hydroxyl and amino or imino groups that enhance the adhesion to many surfaces, particularly those that are smooth textured^32,33^. At the same time, polydopamine exhibits strong UV resistance^26^. At present, the use of polydopamine or SA to improve protection of insect viruses has not been thoroughly studied.

In this study, SeMNPV was encapsulated in calcium alginate microcapsules by atomization and the surface of the microcapsules was modified by chitosan, whey protein and polydopamine. Microcapsules were characterized in terms of structure, morphology and encapsulation efficiency. The pathogenicity and anti-UV properties of the four microcapsules were studied by bioassay. The release of virions by calcium alginate microcapsule (CA-NPV) and polydopamine coated calcium alginate microcapsules (PDA@CA-NPV) in vitro was determined in alkaline solutions. These novel microcapsule materials may have the potential to greatly enhance the efficacy of biological control measures to specifically target lepidopteran pests.

## Materials and methods

### Materials

Sodium alginate (AR, SA), calcium chloride (AR), chitosan (AR), and tris–HCl solution (pH 6.8) were purchased from Aladdin Reagent Co., Ltd. (Shanghai, China). Dopamine hydrochloride (98%) was purchased from Macklin Reagent Co., Ltd., and whey protein (80%) from Shanghai Yuanye Co., Ltd. SeMNPV occlusion bodies (OBs) were provided by the Guangzhou Biological Control Station. The Magnetic Animal Tissue Genomic DNA Kit, Guangzhou Jianlun Biotechnology Co., Ltd; The SYBR Select Master Mix (2X) Quantitative PCR kit, Thermo Fisher Scientific. 2 × high-efficiency PCR mix (including dyes), GETEC; The Thin agarose gel recovery kit, Shanghai Jierui Biotechnology Co., Ltd; The PMD18-T Vector Cloning Kit, TakaRa. Plasmid Mini Preparation Kit (Spin Column Type), Shanghai Jierui Biotechnology Co., Ltd.

### Preparation of SeMNPV microcapsules

Calcium alginate microcapsules were prepared using the method^34^. The inlet temperature of the sprayer, peristaltic pump, and the fan speed was set to 25 °C, 21 mL/min and 4.67 m^3^/min respectively. The SA solution (1.5% w/w) and SeMNPV (2.40 × 10^8^ OBs/mL) with 1.0 mm in diameter are atomized into CaCl_2_ (950 mL 0.2 M) at a distance of 20 cm for 20 min, the deionized water was centrifugally washed and filtered three times. The preparation was stored at room temperature for later use. CA-NPV powder was obtained by freezing in a refrigerator (− 40 °C) and then freeze-drying (Alpha 1–2 LD Plus, Christ, Germany) for 48 h.

### Modification of virus microcapsules

**Figure 1:**
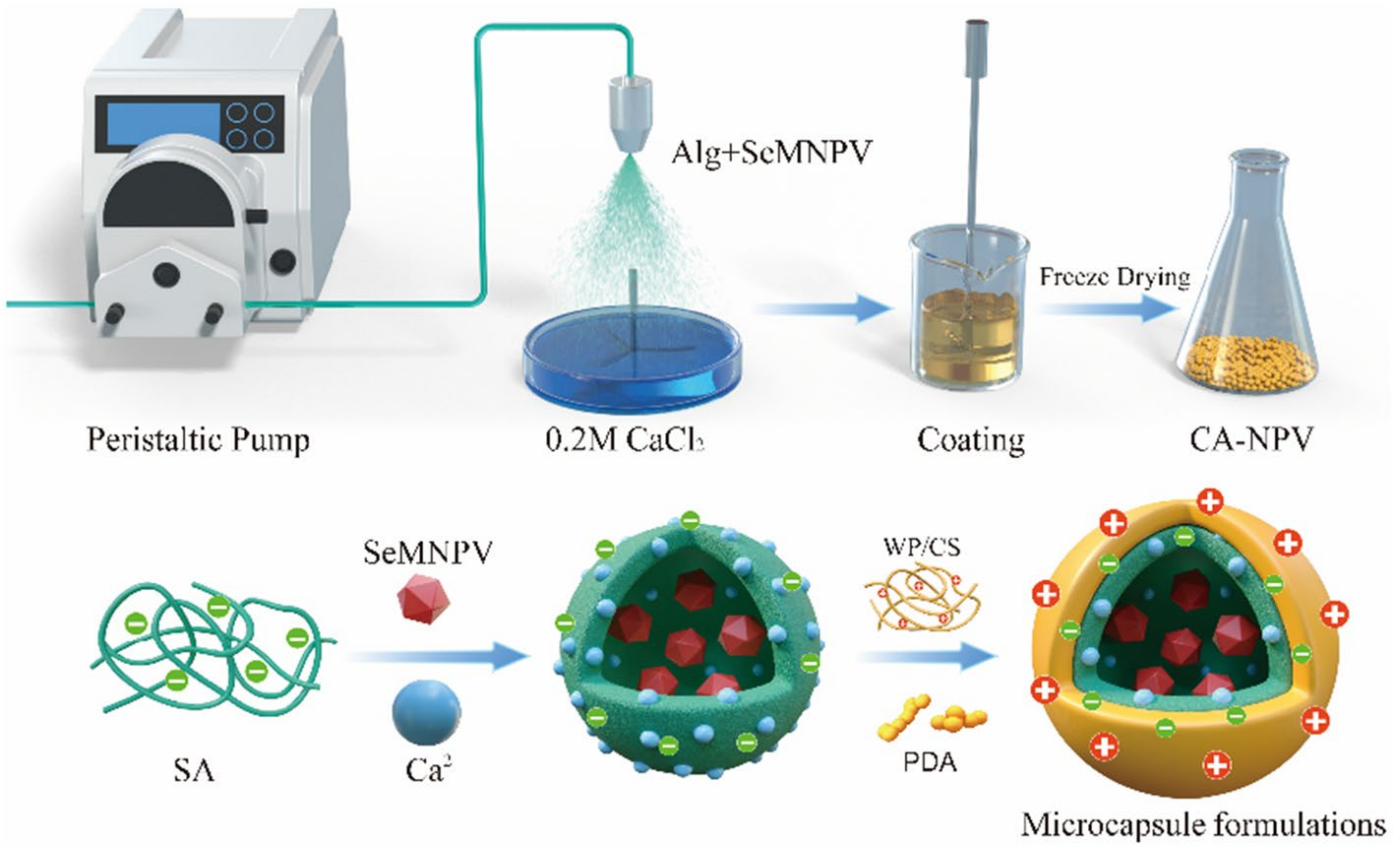
Preparation process of four microcapsule formulations. Solutions of Chitosan and of Whey Protein were prepared. The PDA solution was prepared by adding of dopamine hydrochloride to Tris–HCl buffer, and incubating for 12 h at room temperature. The same quanlity CA-NPV gel was added into the above three solutions, stirred for 40 min, then centrifugally washed thrice. The dried CS@CA-NPV, WP@CA-NPV, and PDA@CA-NPV powders were obtained by freezing in a refrigerator and then freeze-drying for 48 h.

Calcium alginate was modified by chitosan, whey protein and polydopamine, as shown in Fig. 1. SeMNPV was encapsulated in calcium alginate, the modified solution formed a second layer on the calcium alginate. Solutions of Chitosan (1% w/w at pH 5) and of Whey Protein (1% w/w at pH 4.8–5.0) were prepared. The PDA solution was prepared by adding of dopamine hydrochloride (200 mg) to Tris–HCl buffer (100 mL 10 mM at pH 8.5), and incubating for 12 h at room temperature. The same quanlity CA-NPV gel was added into the above three solutions, stirred for 40 min, then centrifugally washed thrice. The dried Chitosan coated calcium alginate (CS@CA-NPV), Whey protein coated in calcium alginate microcapsules (WP@CA-NPV), and PDA@CA-NPV powders were obtained by freezing in a refrigerator (− 40 °C) and then freeze-drying for 48 h. The microcapsule samples were prepared repeatedly and stored at room temperature, and the PDA@CA-NPV was stored in the dark.

### Encapsulation efficiency

The microcapsules were counted using a hemacytometer and light microscopy^35^. The total number (A) observed in the four corners and one center square were tallied. The concentration of virus (C_0_) in filtrate was measured when microcapsules were prepared. Suspensions of SeMNPV with equal numbers of virions were prepared to measure the concentration of virus (C_1_).

Concentrations of SeMNPV preparations were calculated as follows:

1 $${\text{C}}_{0} \left( {{\text{OBs}}/{\text{mL}}} \right) = \frac{A}{80} \times 400 \times 10000 \times 1000$$

The encapsulation efficiency (EE%) was calculated as follows:

2 $${\text{EE}}\% = \left( {{1} - \frac{C0}{{C1}}} \right){{ \times 100\% }}$$

### Fourier transform infrared spectroscopy

Fourier transform infrared (FT-IR) spectra of the samples were obtained on a Spectrum-100 instrument (PerkinElmer Inc., USA), using the KBr pellet method, in the spectral range of 4000–500 cm^−1^^36^.

### X-ray diffraction spectroscopy

Freeze-dried samples (30 mg) were ground and pressed into 1 mm thick circular slices, which were tested by X-ray diffraction (XRD) (Ultima VI, Japan). The test parameters were set as follows: copper target, input = 1.5418 A, 40 kV pipe pressure, 40 mA pipe current, diffraction angle from 2θ to 80°, scanning rate 2°/min^37^.

### Morphological characterization and size distribution

The morphologies of the microcapsules in the samples were determined by fluorescence microscopy (LEICA DM6 B, Germany) and scanning electron microscopy (SEM) (S4800, Hitachi Corporation, Japan). A diluted sample suspension was deposited on a glass slide and observed using a fluorescence microscope. For SEM, the dried sample placed was placed on conductive adhesive and coated with gold using a sputter coater for 45 s. The particle size distributions of the samples were measured by laser diffraction (90Plus, Bruker Analytical Instruments, Germany).

### Contact angle measurements

To determine the contact angles of the samples, diluted microcapsule formulations (20 mg/mL), and SeMNPV OBs (20 mg/mL) were used as a control. The sample (8 µL) was dropped onto the surface of a cucumber leaves. The contact angle was measured using a contact angle meter (Theta, BiolinScientific Co. Ltd., Sweden).

### Virulence assays

Third instar larvae were fasted for 12 h. A diet incorporation method as previously described^38^ was used to investigate the insecticidal activity of SeMNPV OBs and microencapsulated virus samples (CA-NPV, CS@CA-NPV, WP@CA-NPV, PDA@CA-NPV). Deionized water was used as the control treatment. Serial dilutions (2.03 × 10^8^, 2.03 × 10^7^, 2.03 × 10^6^, 2.03 × 10^5^, 2.03 × 10^4^, 2.03 × 10^3^ OBs/mL) of SeMNPV OBs were prepared and microencapsulated. The virus samples were added directly to the artificial diet to achieve the same concentrations as those in the control group. The larvae were then housed at room temperature (n = 30), and each concentration was performed in triplicate (n = 630 larvae total). The pre-experiment fasting protocol helped to ensure that the treated diet was fully consumed within 24 h, at which time the food was replenished. Cumulative mortality rates by day 8 was used to quantify insecticidal activity.

### UV resistance assay

Preliminary bioassays were conducted to determine which five formulations (2.03 × 10^6^ OBs/mL) caused the highest mortality of the *S. exigua* larvae after UVB irradiation. The powders of SeMNPV OBs, CA-NPV, WP@CA-NPV, CS@CA-NPV and PDA@CA-NPV were evenly and thinly spread on weighing paper and exposed for 2 h to UVB output at a distance of 31 cm (20 W, Philips, Poland). The irradiated samples were added to the surface of food, and the 6-day-old second-instar larvae were placed into 24-well plates, one hole and one strip, and each sample was performed in triplicate. Mortality of *S. exigua* larvae was assessed daily until all larvae had died or the study was terminated. Cumulative mortality was calculated.

### In vitro virus release

The in vitro release tests were conducted by qPCR, using Sodium hydroxide solution (pH 10). Samples of the receiving buffer were withdrawn at different time intervals and the viral genome copies were quantified by qPCR. The same volume of fresh receiving buffer was added to replace the volume of the withdrawn aliquots.

Quantitative PCR was carried out using the SYBR Select Master Mix (2X) (ABI, Germany) in Applied Biosystems StepOnePlus Real-Time System (Germany). The purified polyhedrosis virus sample (20 μL) was placed in the enzyme-free centrifuge tube and DNA was extracted using the DNA Viral Genome Extraction Kit (Solarbio). Primers and probes were designed with Beacon Designer and supplied by TIANYIHUIYUAN. For SeMNPV, SA-NPV and PDA-NPV, 10 nM of each primer (forward 5′-AGCCTTGGGTCGCACTTACG-3′; reverse 5′-ACTTTTGGTTTTTGCCGGGT-3′) were used in a 30 μL reaction volume. The product was detected and recovered on a 1% agarose gel. After the target fragment was excised and eluted, it was cloned into the PMD18-T vector and transferred into *E. coli*. Natural competence, screened by galactosidase activity (blue/white colony selection) and identified by colony PCR, the positive transformants were sent to the Wuhan Tianyi Company for sequencing. The accuracy of the standard curve was verified by melting curve analysis. A tenfold serial dilution series of the plasmid, ranging from 10^3^ to 10^9^ copies/μl, was used to construct the standard curves for SeMNPV DNA. The concentration of the plasmid was measured using a fluorometer and the corresponding copy number was calculated using the following equation^39,40^:

3 $$\mathrm{DNA } \, (\mathrm{copy})=\frac{6.02\times {10}^{23}(\mathrm{cop}y/mol)\times \mathrm{DNA \, amount}(\mathrm{g}) }{DNA \, length(dp)\times 660(\mathrm{g}/\mathrm{mol}/\mathrm{dp})}$$

CT values in each dilution were measured in duplicate using a real-time QPCR with the plasmid to generate the standard curves for SeMNPV DNA. The CT values were plotted against the logarithm of their initial template copy numbers. Each standard curve was generated by a linear regression of the plotted points. From the slope of each standard curve, PCR amplification efficiency (E) was calculated according to the equation^41^:

4 $${\mathrm{E}= 10}^{\frac{-1}{\mathrm{slope}}}-1$$

Absolute quantification methods^42^ were simultaneously used to quantify the plasmid copy. Absolute quantification determines the exact copy concentration of target gene by relating the CT value to a standard curve^43^.

### Statistical analysis

All measurements were performed in triplicate. The results obtained were presented as means. Figure were mapped using the Origin 9.10.00 software (https://pan.baidu.com/s/1JURZ0VdxshFeZa5MzsUhaQ). Table were mapped using the Microsoft Office 2016 (https://pan.baidu.com/s/1CPQLjpE5FRXSRvPm-u_Ywg). Log transformed virus test concentrations (OBs/mL) were regressed on cumulative mortality data using Probit analysis to estimate LC_50_ values at 95% confidence intervals^44^ using SPSS Base 24.0.0.0 software (https://pan.baidu.com/s/1s7hsJaBCKqoTuWTBzH_5Zg).

## Results

### Infrared spectrum analysis

**Figure 2:**
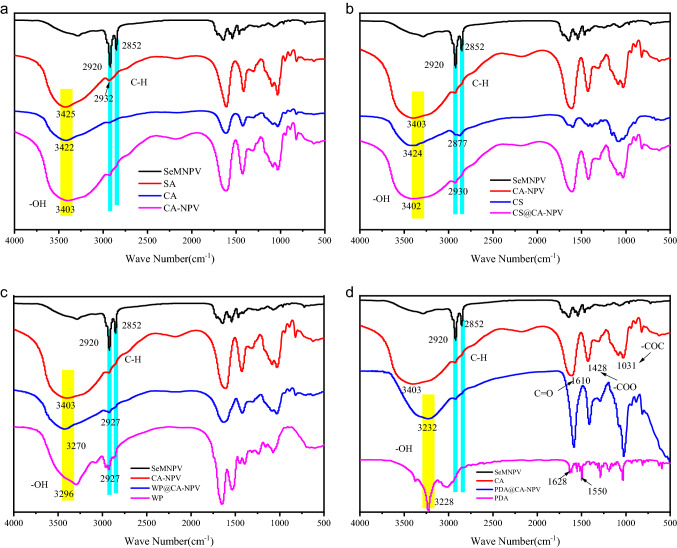
IR spectra of SeMNPV, CA-NPV, CS@CA-NPV, WP@CA-NPV, PDA@CA-NPV, and their raw materials. IR spectra of the samples were obtained on a Spectrum-100 instrument, using the KBr pellet method, in the spectral wavelength's range of 4000–500 cm^−1^. An abundance of hydroxyl groups were represented at 3000–3500 cm ^−1^. SeMNPV produced two distinct bands at 2920 cm^−1^ and 2852 cm^−1^, which correspond to the asymmetric and symmetric stretching vibrations of methylene C–H, respectively. In Fig. 2d, the absorption bands at 1520, 1615, and 3420 cm^−1^ corresponded to the shearing vibration of N–H in the amide group, aromatic rings, and the –OH groups in catechol, respectively. The PDA@CA-NPV produced the absorbance peaks at 1610, 1428 and 1031 cm^−1^ were attributed to C=O stretching vibrations. asymmetric vibrations of –COO, and alkyl C–O–C deformation vibrations of CA-NPV.

Preparations of SeMNPV, CA-NPV, CS@CA-NPV, WP@CA-NPV, PDA@CA-NPV, and their raw materials were characterized by FT–IR spectroscopy. Because of the polysaccharide or protein-like structure of SeMNPV and microcapsule preparations, many characteristic peaks with similar functional groups were observed. An abundance of hydroxyl groups were represented at 3000–3500 cm^−1^^45^. As shown in Fig. 2, the absorption peak of CA-NPV was wider than that of CA, which indicates that hydrogen bonds formed between CA-NPV and SeMNPV. Compared with CS@CA-NPV and WP@CA-NPV, the absorption peak of CA-NPV was wider, which is indicative of more hydrogen bonds. These hydrogen bonds may be formed by the interaction between the amino group of chitosan and the carboxyl group of calcium alginate, and between the amino group of whey protein and the carboxyl group of calcium alginate. In Fig. 2d, the absorption bands at 1520, 1615, and 3420 cm^−1^ corresponded to the shearing vibration of N–H in the amide group, aromatic rings, and the -OH groups in catechol, respectively. These data suggest that successful polymerization of dopamine occurred^46^. The PDA@CA-NPV produced similar FT-IR absorbance patterns to the pristine CA-NPV, and the absorbance peaks at 1610, 1428 and 1031 cm^−1^ were attributed to C=O stretching vibrations^47^. asymmetric vibrations of –COO^48^, and alkyl C–O–C deformation vibrations of CA-NPV^49^. These observations further confirm the presence of CA-NPV in the PDA@CA-NPV. Additionally, SeMNPV produced two distinct bands at 2920 cm^−1^ and 2852 cm^−1^, which correspond to the asymmetric and symmetric stretching vibrations of methylene C-H, respectively.

### X-ray diffraction analysis

**Figure 3:**
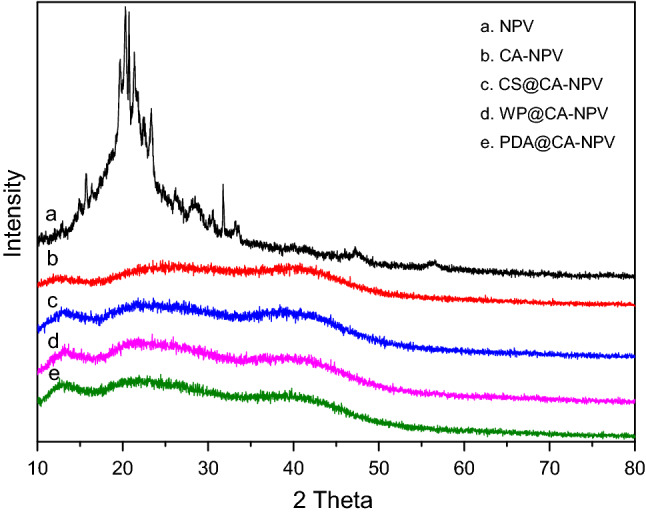
XRD patterns of SeMNPV and microencapsulated preparations. Freeze-dried samples were ground and pressed into 1 mm thick circular slices, which were tested by XRD. The test parameters were set as follows: copper target, input = 1.5418 A, 40 kV pipe pressure, 40 mA pipe current, diffraction angle from 2θ to 80°, scanning rate 2°/min. the SeMNPV has a sharp diffraction peak and a flat baseline, which is expressed as a crystalline state. The diffraction curve of the polysaccharide contained only a few small, and no sharp, peaks. No obvious diffraction peak for the PDA was observed.

As can be seen in Fig. 3, the SeMNPV has a sharp diffraction peak and a flat baseline, which is expressed as a crystalline state. In the baculovirus-Sf9 expression system, virions are occluded with a crystalline polyhedrin matrix, representing a functional biological crystallization system characteristic of the virus^50^. The diffraction curve of the polysaccharide contained only a few small, and no sharp, peaks. This is indicative of the polysaccharide precipitating as an amorphous material under vacuum freeze drying and vacuum-drying processes^51^. No obvious diffraction peak for the PDA was observed, which may be responds for the relatively thin layer and amorphous crystallinity of the PDA prepared using this polymerization method^52^. It can be seen that there are almost no fully exposed SeMNPV OBs on the surface of the four microcapsule preparations.

### Morphological characterization analyses

To examine the morphology and structural composition of the polymer-virus mixtures, light and scanning electron (SEM) microscopy were used to visualize the various formulations.

**Figure 4:**
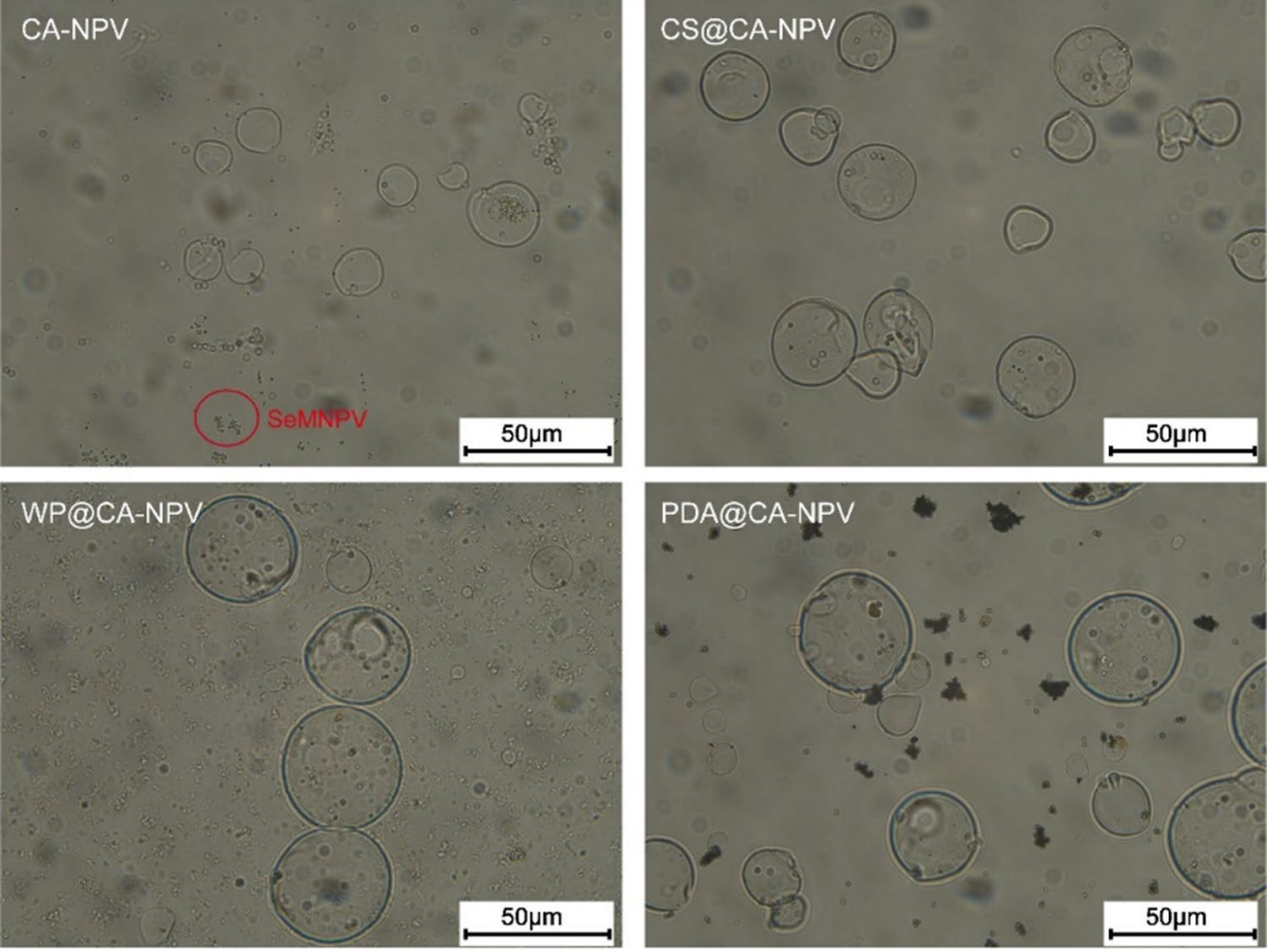
Surface morphology of microencapsulated preparations under light microscopy. A diluted sample suspension was deposited on a glass slide and observed using a fluorescence microscope.

**Figure 5:**
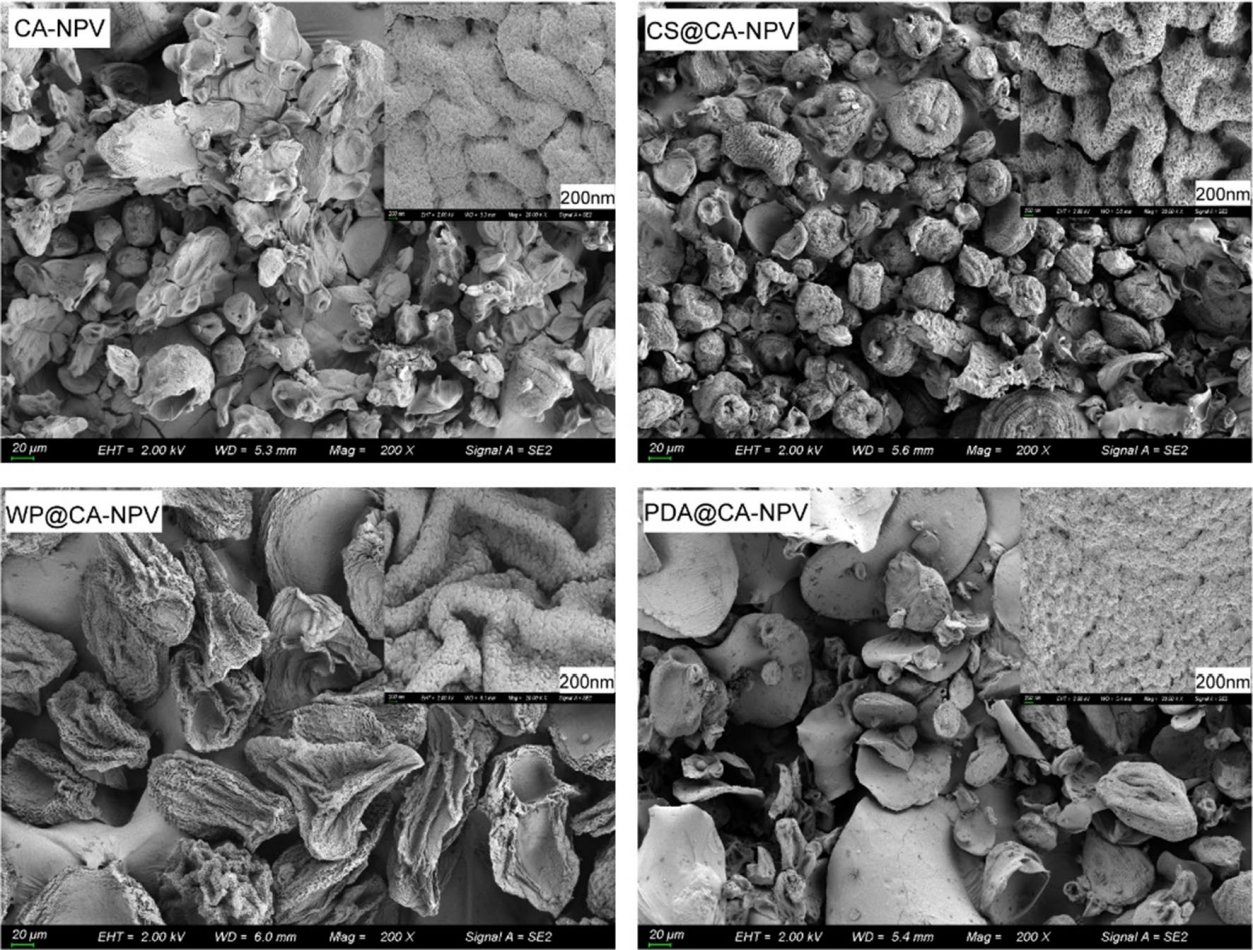
Surface morphology of microencapsulated preparations under SEM. The dried sample placed was placed on conductive adhesive and coated with gold using a sputter coater for 45 s. In the CA-NPV samples, shriveling due to water evaporation during the sample preparation process was observed. Observations of CS@CANPV and WP@CA-NPV revealed that the electrostatic self-assembled microcapsules were more complete and more spherical than single-layer microcapsules. The 1,2-dihydroxybenzene and amino groups in the structure of dopamine self-oxidized and polymerized on almost any solid surface to form a dopamine coating that adhered tightly to the surface of the microcapsule.

Under the light microscope, that the four types of unwashed microcapsules were well dispersed and had no obvious agglomeration Fig. 4. SeMNPV OBs can be seen inside and on the surface of the microcapsules. The morphology of microencapsulated powders was observed by scanning electron microscopy (SEM), as shown in Fig. 5. In the CA-NPV samples, shriveling due to water evaporation during the sample preparation process was observed. Observations of CS@CA-NPV and WP@CA-NPV revealed that the electrostatic self-assembled microcapsules were more complete and more spherical than single-layer microcapsules. The 1,2-dihydroxybenzene and amino groups in the structure of dopamine self-oxidized and polymerized on almost any solid surface to form a dopamine coating that adhered tightly to the surface of the microcapsule. As a result, a plurality of microcapsules appears adhered together to form a sheet-like microcapsule. When magnified up to 5,000x, all four microcapsules exhibited pores, which facilitated the entry of alkaline liquids, calcium alginate disintegration, and release of baculovirus virions.

### Size distribution analysis

**Figure 6:**
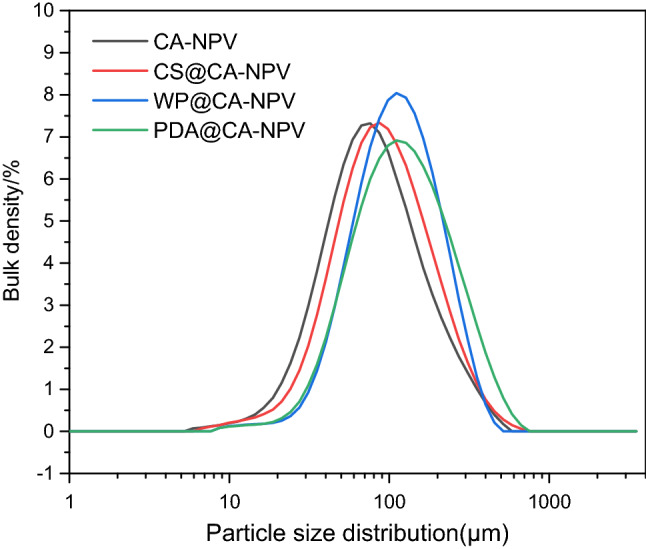
Particle size distribution of microencapsulated preparations. The particle size distributions of the samples were measured by laser diffraction. The corresponding diameters of 90% (DV(90)) were 215 μm, 236 μm, 252 μm and 313 μm, respectively.

The size distributions of CA-NPV, CS@SA-NPV, WP@SA-NPV, and PDA@SA-NPV are shown in Fig. 6. The results indicated that the corresponding diameters of 90% (D_V_(90)) were 215 μm, 236 μm, 252 μm and 313 μm, respectively. As seen in Fig. 5. showed that the particle size of the modified microcapsules was larger than that of the original microcapsules. The chitosan and whey protein solutions were positively charged and self-assembled to form the second shell in CA-NPV. The PDA caused several CA-NPV microcapsules to adhere, forming massive microcapsules. Therefore, the size of PDA@CA-NPV microcapsules was larger than that of electrostatic self-assembled microcapsules.

### Contact angle measurements analysis

**Figure 7:**
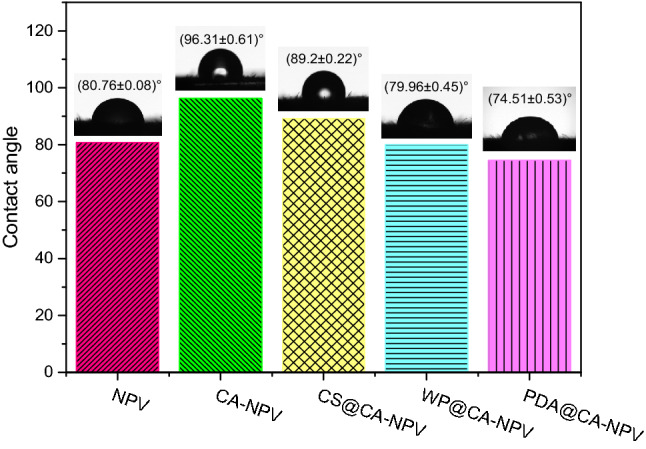
Contact angle of SeNPV OBs and four microencapsulated preparations. To determine the contact angles of the samples, diluted microcapsule formulations, and SeMNPV OBs were used as a control. The sample was dropped onto the surface of a cucumber leaves. The contact angle was measured using a contact angle meter. The contact angles of SeMNPV and microcapsule preparations varied, from small to large, in order as follows: PDA@CA-NPV < WP@CA-NPV < SeMNPV < CS@CA-NPV < CA-NPV.

Assuming that more active ingredient residue on the leaf surface would result in higher pest mortality, the spreading effect of each sample on the leaf surface was tested. As shown in Fig. 7, the contact angles of SeMNPV and microcapsule preparations varied, from small to large, in order as follows: PDA@CA-NPV < WP@CA-NPV < SeMNPV < CS@CA-NPV < CA-NPV. Although the surface materials of each group were different, the contact angles were all less than 110°, and the leaf surface was easily wetted^53,54^. Foliage is covered with a waxy layer composed primarily of negatively charged fatty acids and alcohols. The surface layer of polydopamine contains a large number of 1,2-dihydroxybenzene and amino groups, which can form strong hydrogen bonds and have electrostatic interactions with the leaf surface^55^. Thus, the microcapsules can be easily wetted and distributed over the leaf surface. The smaller the contact angle, the greater the retention and the wider the spread of the droplet over the leaf surface. Since WP@CA-NPV and SeMNPV both consist of proteinaceous surfaces; the contact angles were similar in size. Because chitosan contains a large number of polar groups such as hydroxyl and amino groups in its molecular chain, the adhesion between positively charged amino groups and negatively charged fatty acids and aliphatic alcohols can be strengthened by electrostatic interaction. The aqueous solution of CS@CA-NPV microcapsules exhibited good wettability on the leaf surface. However, the amino group in chitosan reacted with the carboxyl group in calcium alginate to form a composite membrane, which resulted in the reduction of polar groups. The surface negative charge of CA-NPV microcapsules and the negative charge of the cucumber leaves repel each other, resulting in a maximum contact angle. Therefore, PDA@CA-NPV has the minimum contact angle and the best wettability.

### Insecticidal activity analysis

Table 1 shows the results of the insecticidal activity tests for each formulations. The LC_50_ values for the PDA@CA-NPV, WP@CA-NPV, CS@CA-NPV and CA-NPV microcapsules were 1.2, 10.6, 10.7 and 11.5 fold that of unformulated SeMNPV OBs, respectively. The LC_50_ of polydopamine-modified microcapsules is second only to virus alone. Dopamine, the precursor for melanin, is produced by the epidermis at the end of the molt^56^. Hiruma found that epidermal dopamine is incorporated into the cuticle beginning 3 h before ecdysis, and peaks 3 h after ecdysis^57^. Probably due to the existence of PDA, PDA@CA-NPV was the highest insect activity of the four microcapsule preparations. It is well known that insect larvae contain chitin and protein^58^, and are potential sources of chitin and chitosan^59–61^. Similarly, the larval “defense system” may not regard CS@CA-NPV or WP@CA-NPV as foreign material. The unmodified CA-NPV may therefore be identified and partially excreted in the faeces, resulting in the highest LC_50_.

**Table 1:** Regression equation, correlation coefficients, and LC_50_ between the SeMNPV and microcapsule formulations and the mortality of 3^rd^ instar larvae.

Treatment	Regression equation	Slope ± SE	LC_50_ (PIBs/mL)	95% confidence interval (PIBs/mL)	X^2^
SeMNPV	y = 0.8628 + 0.7539x	0.630–0.877	3.07 × 10^5^	1.08 × 10^5^–8.70 × 10^5^	0.986
CA-NPV	y = 1.7632 + 04943x	0.303–0.685	3.54 × 10^6^	1.34 × 10^6^–9.33 × 10^7^	0.928
CS@CA-NPV	y = 1.7300 + 0.5017x	0.300–0.703	3.30 × 10^6^	1.27 × 10^6^–8.57 × 10^7^	0.923
WP@CA-NPV	y = 2.3248 + 0.4109x	0.261–0.561	3.24 × 10^6^	1.07 × 10^6^–9.81 × 10^6^	0.935
PDA@CA-NPV	y = 0.6429 + 0.7818x	0.572–0.992	3.74 × 10^5^	1.41 × 10^5^–9.94 × 10^5^	0.964

The two groups with the highest lethal effect on larvae were SeMNPV and PDA@CA-NPV statistically significant differences in mortality were observed between CA-NPV, CS@CA-NPV, and WP@CA-NPV.

### UVB resistance analysis

**Figure 8:**
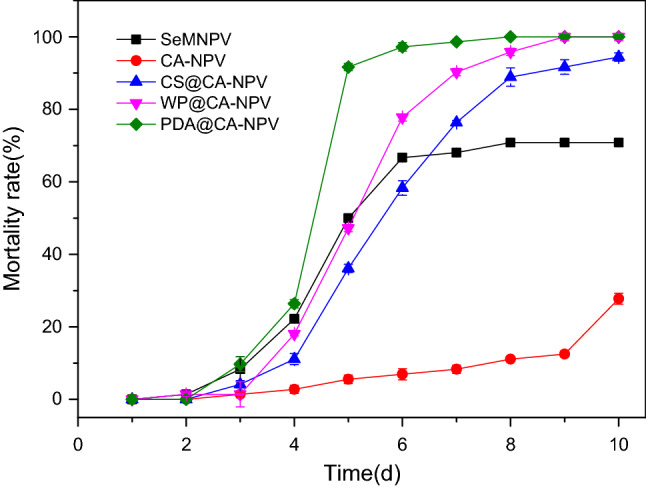
Mortality of SeMNPV and microcapsule formulations following UVB treatment for 2 h, in 2nd instar larvae. The powders of SeMNPV OBs, CA-NPV, WP@CA-NPV, CS@CA-NPV and PDA@CA-NPV were evenly and thinly spread on weighing paper and exposed for 2 h to UVB output at a distance of 31 cm. The irradiated samples were added to the surface of food, and the 6-day-old second-instar larvae were placed into 24-well plates, one hole and one strip, and each sample was performed in triplicate. A small number of deaths occurred in each treatment on the third day after exposure, with mortality rates of 8.3% and 9.7% in the SeMNPV and PDA@CA-NPV treated groups, respectively. On day 5, a large number of deaths occurred, with 50% mortality being observed in the SeMNPV infected group. Also on day 5, the mortality of PDA@CA-NPV treated insects was 91.7%. Over time, on the 8th day, PDA@CA-NPV produced 100% mortality. The mortality of WP@CA-NPV and CS@CA-NPV groups was 95.8% and 88.9%, respectively. The mortality of SeMNPV infected larvae reached to 70.8%.

**Figure 9:**
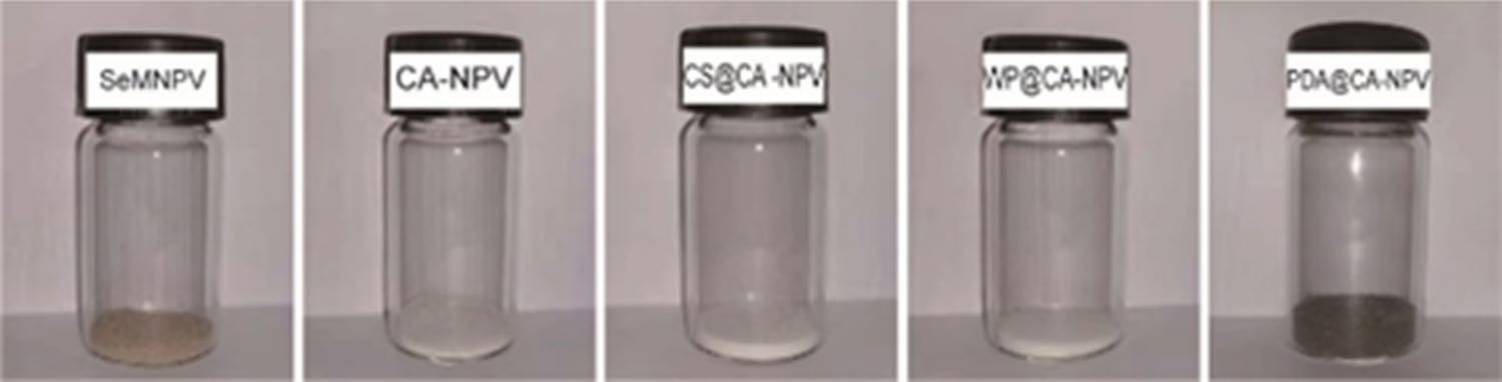
Microcapsule samples after freeze-drying were stored at room temperature. The microcapsules used for UV resistance experiments were at room temperature stored for 8 months in the glass bottle.

Figure 8 shows the mortality of the second instar *S. exigua* larvae caused by SeMNPV OBs and the microcapsules at a viral titer of diet (2.03 × 10^6^ OBs/mL) after 2 h of UVB irradiation. A small number of deaths occurred in each treatment on the third day after exposure, with mortality rates of 8.3% and 9.7% in the SeMNPV and PDA@CA-NPV treated groups, respectively. On day 5, a large number of deaths occurred, with 50% mortality being observed in the SeMNPV infected group. Also on day 5, the mortality of PDA@CA-NPV treated insects was 91.7%. Over time, on the 8th day, PDA@CA-NPV produced 100% mortality. The mortality of WP@CA-NPV and CS@CA-NPV groups was 95.8% and 88.9%, respectively. The mortality of SeMNPV infected larvae reached to 70.8%, indicating WP@CA-NPV and CS@CA-NPV were capable of protecting SeMNPV from UVB irradiation. However, the mortality observed in the CA-NPV treated group was only 11.11%. This decrease in mortality may be the result of ineffective protection of the virus by the microcapsules from UVB irradiation, and potentially because of a delayed release of the virus by the CA-NPV microcapsules. The results showed that PDA@CA-NPV had the best anti-uv performance. As shown in Fig. 9, the microcapsules used for UV resistance experiments were at room temperature stored for 8 months in the glass bottle, and the loss of activity was negligible. Variance analysis was performed on the final experimental results, as shown in Table 2. A total of five groups were repeated three times for each group. Analysis with SPSS Software showed that the number of days had a significant effect on the mortality.

**Table 2:** Ten days mortality values subjected to ANOVA.

	SeMNPV	CA-NPV	CS@CA-NPV	WP@CA-NPV	PDA@CA-NPV
F value	88.679	13.355	136.04	88.679	450.285
P value	P = 0.0001	P = 0.001	P = 0.0001	P = 0.001	P = 0.001

### In vitro virus release analysis

**Figure 10:**
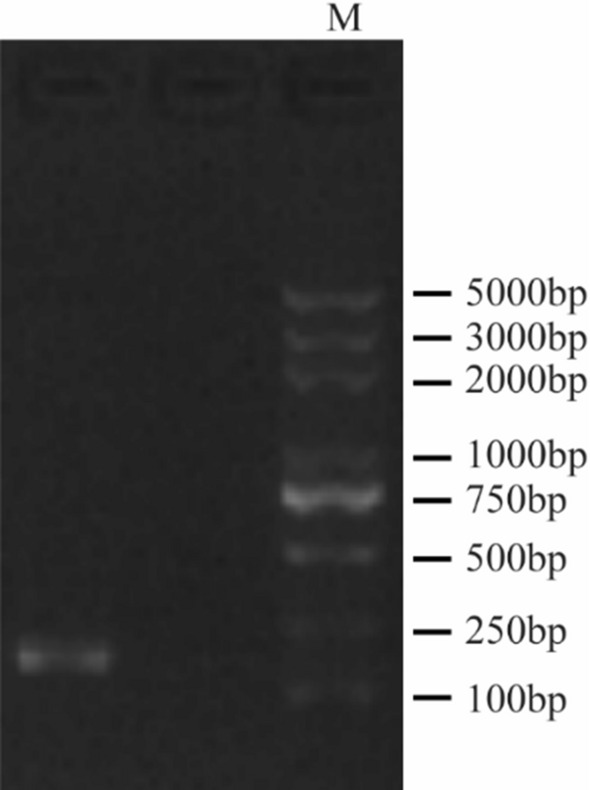
Identification of PCR amplicon size by gel electrophoresis. The 5 mL of the amplified products were used for 1% agarose gel electrophoresis. The size of the extracted fragment was between 100 and 250 bp.

The specificity of the amplified products was confirmed by purifying the PCR products from agarose gels using a Gel Extraction Kit (Takara Bio, Otsu, Shiga, Japan). Primers that gave clear specific bands without any nonspecific amplification (as observed using electrophoresis) were selected. As shown in Fig. 10, the results of electrophoresis showed that the size of the extracted fragment was between 100 and 250 bp, which was consistent with the expected amplicon size of 182 bp targeted by the primers as designed.

Next, the fragments were ligated into the pMD18-T vector, cloned into *E. coli* and amplified for subsequent sequencing. The data were analyzed using Bioedit (7.0.7.1), which confirmed that the amplified product was from the targeted SeMNPV Polyhedrosis protein gene fragment. The sequence specificity was further verified by a blastp search of the NCBI database, which showed considerable similarity among the targeted viral sequences.

The genomic DNA of SeMNPV was amplified using qPCR with specific primers and target DNA in tenfold serial dilutions (10^8^–10^3^). The assay yielded an average amplification efficiency of 85–105%, and the amplification curves were smooth and orderly. The melting curve analysis produced a single peak corresponding to 82.88 °C, which is indicative of specific amplification. The standard curve equation was Y = − 3.2152X + 39.025, R^2^ = 0.999.

**Figure 11:**
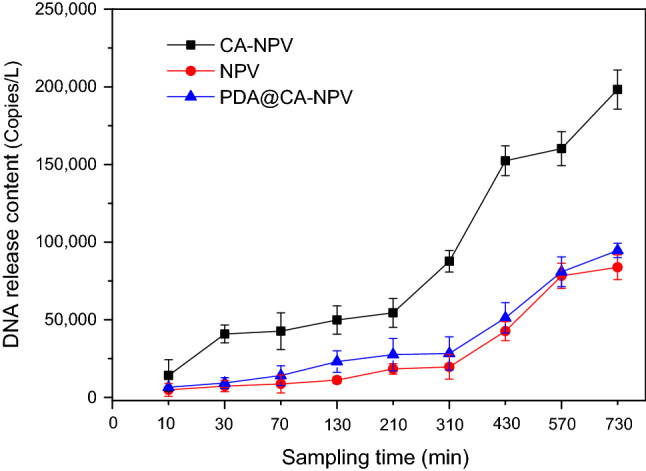
Release of genomics DNA from SeMNPV OBs and CA-NPV and PDA@CA-NPV encapsulated formulations at in sodium hydroxide solution. The standard curve was obtained by diluting the standard sample with 6 gradients: 10^8^, 10^7^, 10^6^, 10^5^, 10^4^, 10^3^, and taking the CT value as the horizontal coordinate and the concentration logarithm of dilution multiple as the vertical coordinate. Samples of the receiving buffer were withdrawn at different time intervals and the viral genome copies were quantified by qPCR. the DNA concentration of CA-NPV, SeMNPV, PDA@CA-NPV was 22,736 copies/L, 4897 copies/L, and 6519 copies/L, respectively, in NaOH (pH 10) for 10 min. CA-NPV and PDA@CA-NPV released the contained virus rapidly after 310 min. The slope of the release curve increased dramatically, and the release rate decreased gradually from 570 to 730 min.

According to the standard curve, the same primers and conditions were used for qPCR amplification, in triplicate. Standard plasmid 96 well plate was used for positive control and error correction, and NTC was used for negative control. Because PDA@CA-NPV had the strongest the UVB effect in anti-UV test, the in vitro sustained-release effect of PDA@CA-NPV was tested. The results showed that DNA concentration increased with time. As shown in Fig. 11, the DNA concentration of CA-NPV, SeMNPV, PDA@CA-NPV was 22,736 copies/L, 4,897 copies/L, and 6,519 copies/L, respectively, in NaOH (pH 10) for 10 min. CA-NPV and PDA@CA-NPV released the contained virus rapidly after 310 min. the slope of the release curve increased dramatically, and the release rate decreased gradually from 570 to 730 min. Microencapsulated SeMNPV did not change the release pattern of the virus under alkaline conditions, and also confirmed the successful encapsulation of the virus. Variance analysis was performed on the final experimental results, as shown in Table 3. A total of three groups were repeated three times for each group. Analysis with SPSS Software showed that release time had a significant effect on the mortality.

**Table 3:** DNA release content with sampling time subjected to ANOVA.

	CA	SeMNPV	PDA@CA-NPV
F value	F = 4.446	F = 17.312	F = 4.799
P value	P = 0.02	P = 0.0001	P = 0.003

## Conclusions

In this study, CA-NPV microcapsules were prepared by spray method, then CA-NPV was modified by CS, WP or PDA treatment. The results indicated that the virus was contained within the microcapsules. Compared with CA-NPV, the modified microcapsules were more complete structure and spherical. By modifying CA-NPV with CS, WP or PDA, the anti-UV property of SeMNPV can be improved, which is expected to solve the problem of inactivation of virus by UV irradiation in practical application. After UVB irradiation, the highest lethal rate, 100% and the shortest lethal time of PDA@CA-NPV were obtained. In contrast to previously reported studies, alkaline controlled release vectors were more effective, and in vitro release tests under alkaline conditions showed that the microencapsulated virus did not change the release pattern of virions. Therefore, the use of microencapsulated virus can provide more synergist, sex pheromone mixed possibility, shorten the killing time, improve the efficiency of pest control, enhance the practicality of biological control strategies.

## Supplementary Information

Supplementary Information 1.

Supplementary Information 2.

Supplementary Information 3.

Supplementary Information 4.

Supplementary Information 5.

Supplementary Information 6.

Supplementary Information 7.

## Data Availability

The datasets used and analysed during the current study available from the corresponding author on reasonable request. All data generated or analysed during this study are included in this published article and its Supplementary information files.
