# Supplementary figures and images for: Study on physical properties of four pH responsive Spodoptera exigua multiple nucleopolyhedrovirus (SeMNPV) microcapsules as controlled release carriers
Source: Sci Rep. 2022 Dec 19;12:21873. doi: 10.1038/s41598-022-26317-5 (PMC9763348; doi:10.1038/s41598-022-26317-5)

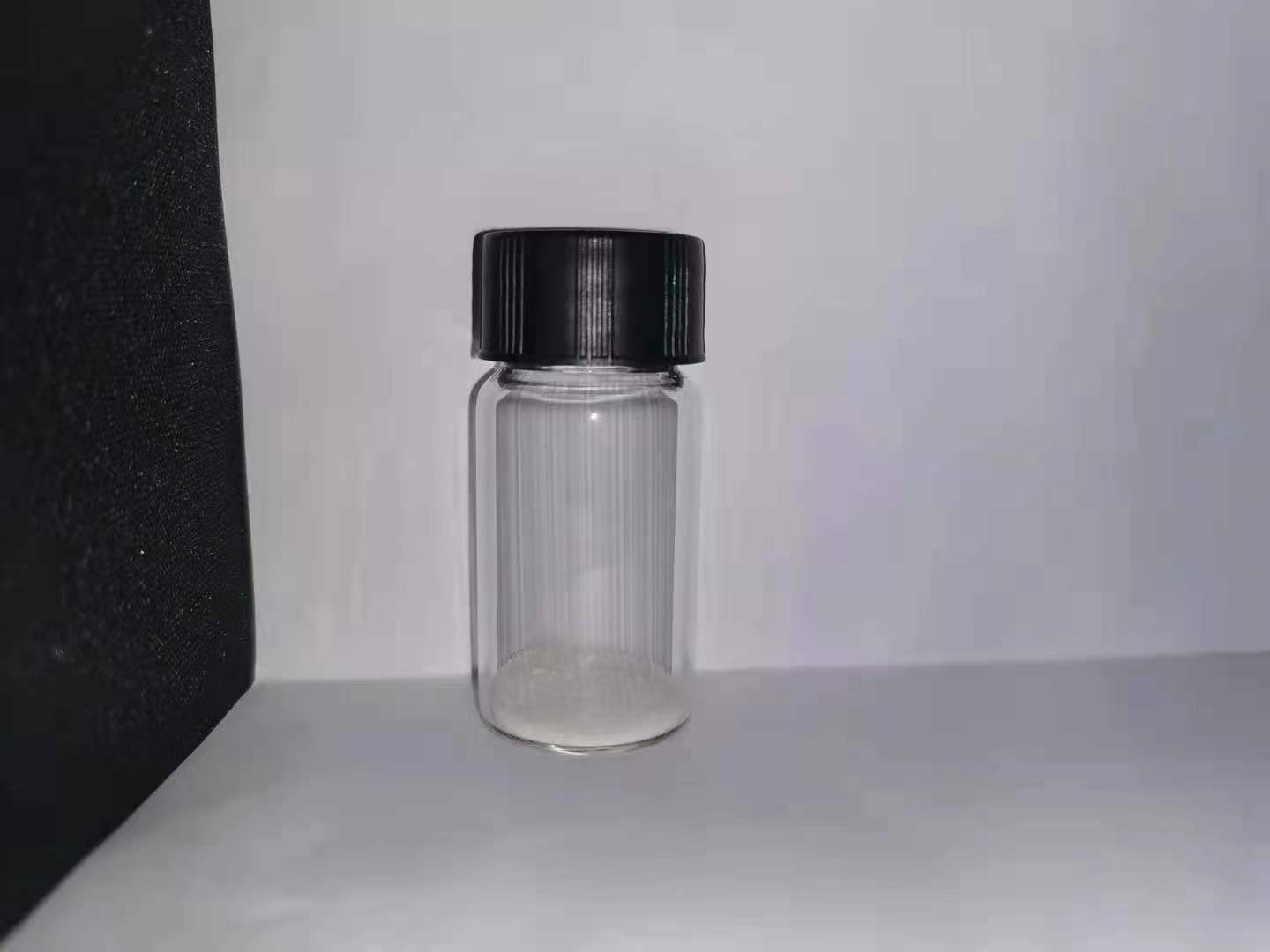

Supplement: Supplementary file 1 — Supplementary Information 1. [file 41598_2022_26317_MOESM1_ESM.jpg]

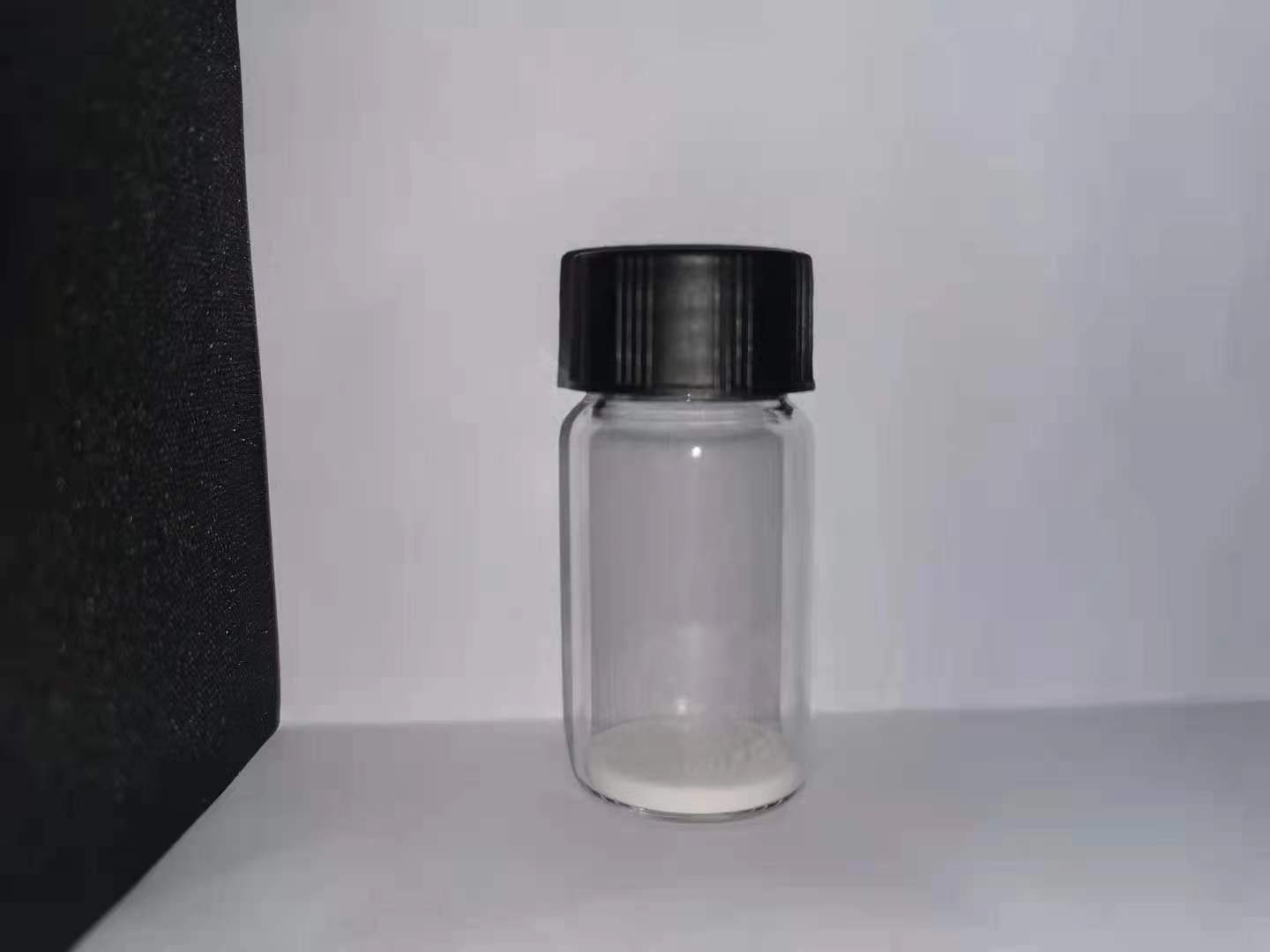

Supplement: Supplementary file 2 — Supplementary Information 2. [file 41598_2022_26317_MOESM2_ESM.jpg]

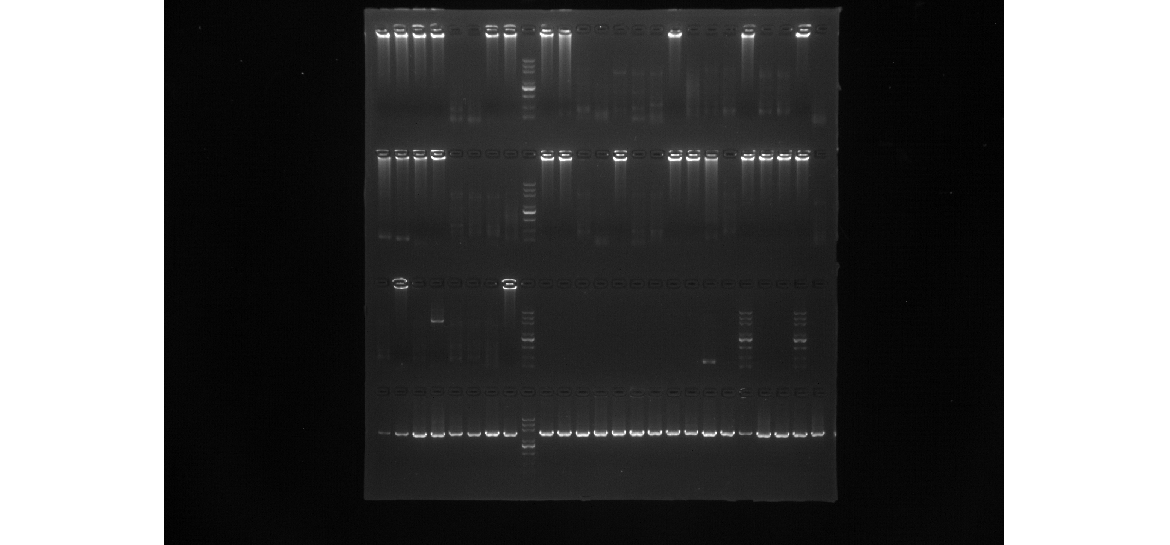

Supplement: Supplementary file 3 — Supplementary Information 3. [file 41598_2022_26317_MOESM3_ESM.jpg]

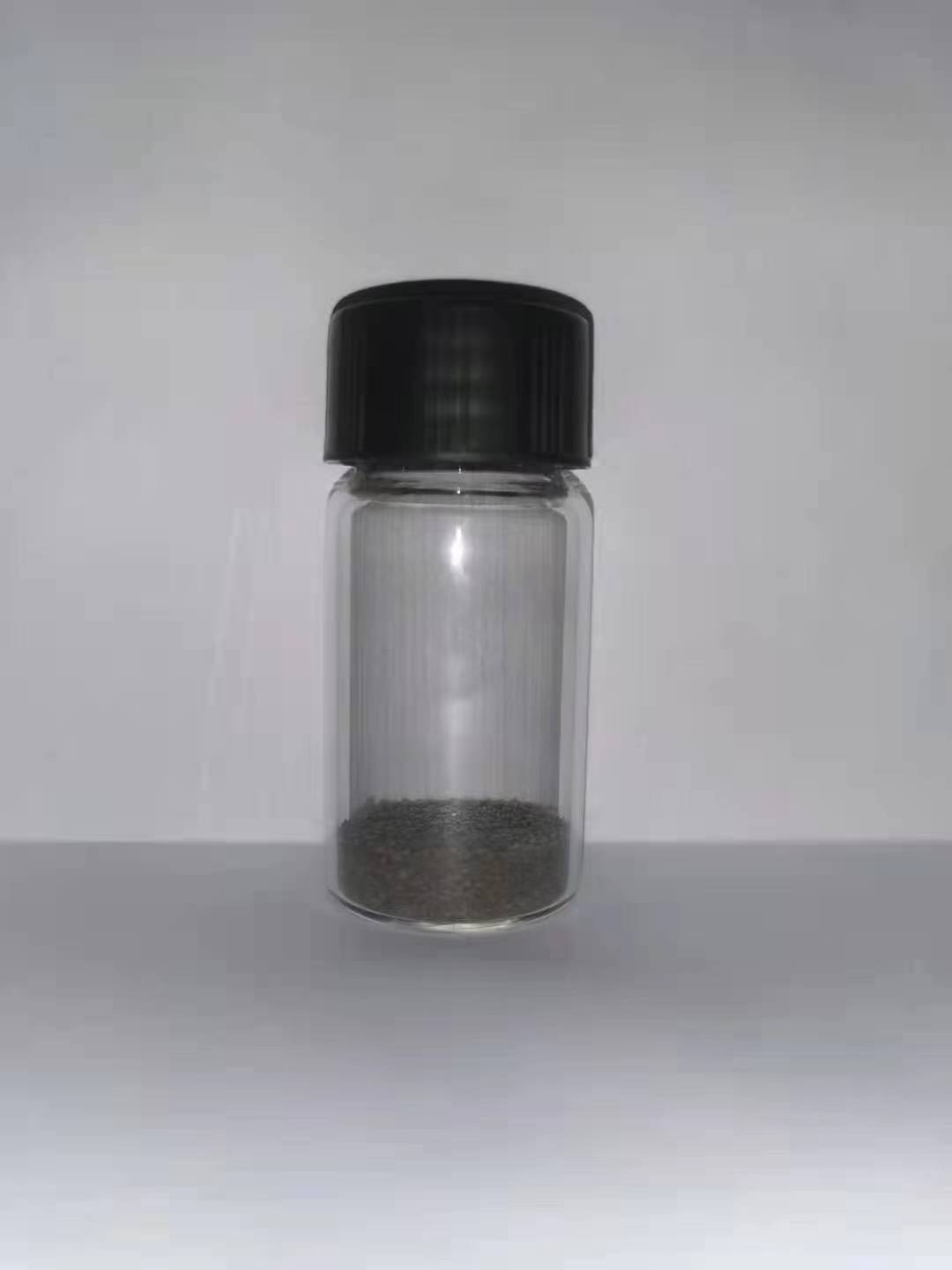

Supplement: Supplementary file 4 — Supplementary Information 4. [file 41598_2022_26317_MOESM4_ESM.jpg]

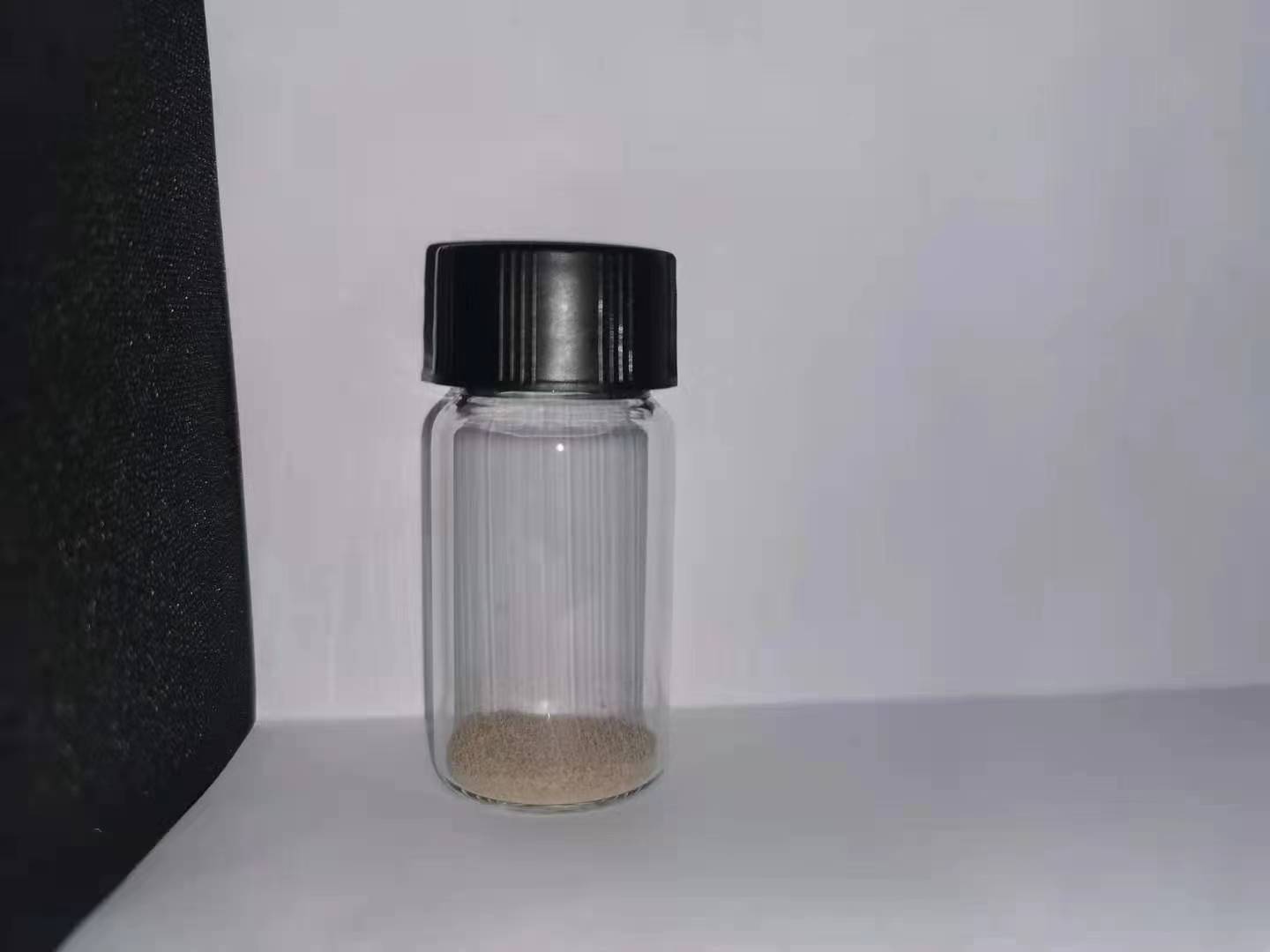

Supplement: Supplementary file 5 — Supplementary Information 5. [file 41598_2022_26317_MOESM5_ESM.jpg]

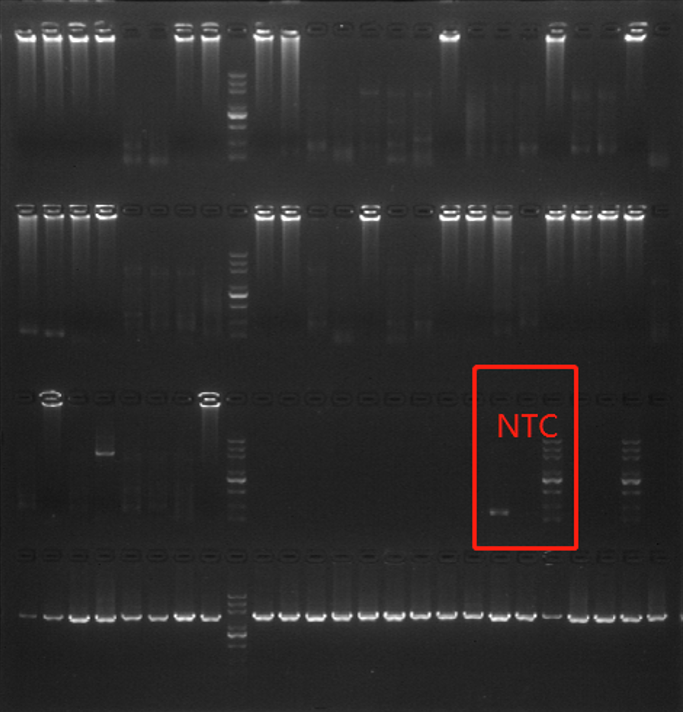

Supplement: Supplementary file 6 — Supplementary Information 6. [file 41598_2022_26317_MOESM6_ESM.png]

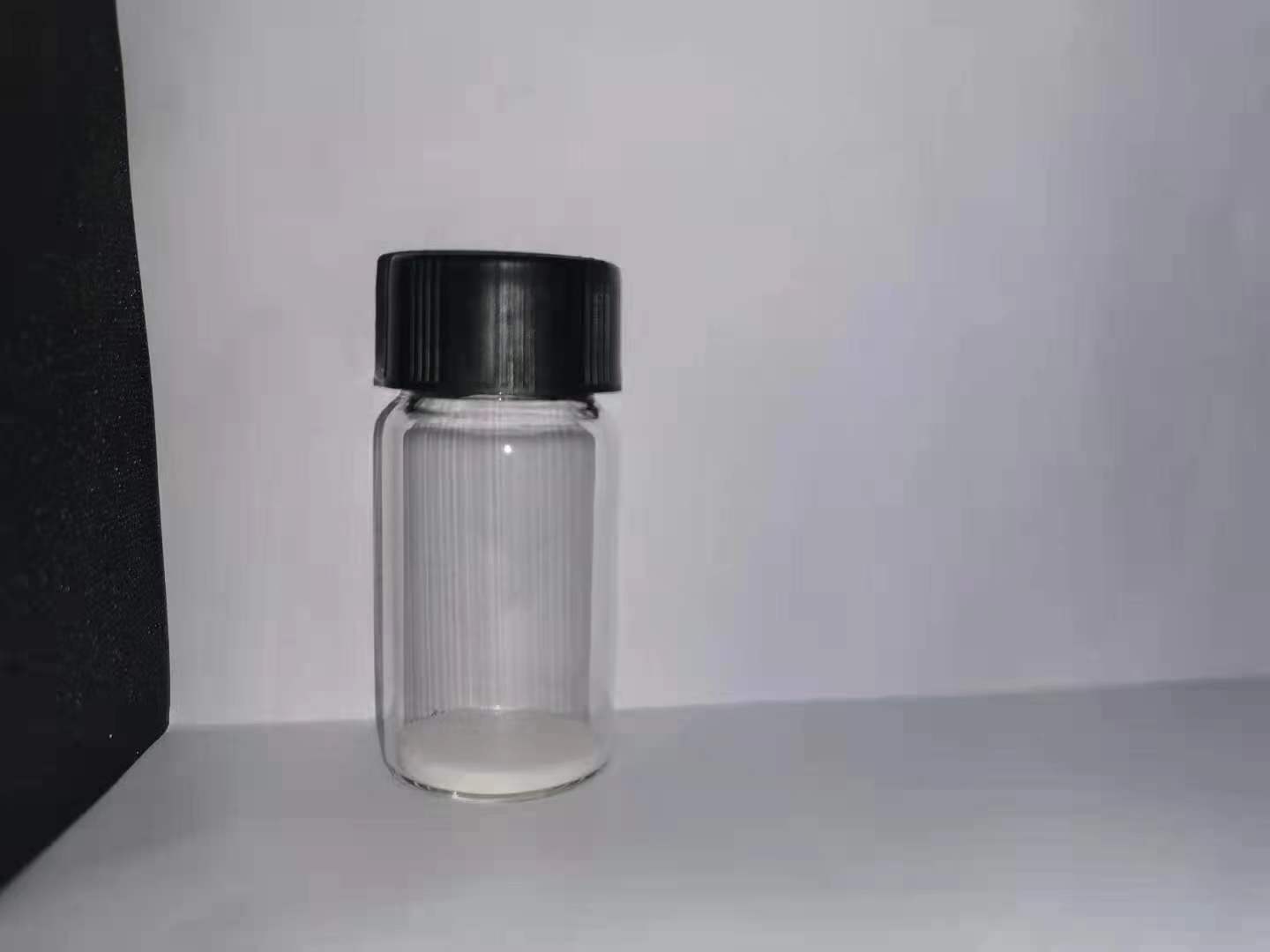

Supplement: Supplementary file 7 — Supplementary Information 7. [file 41598_2022_26317_MOESM7_ESM.jpg]
